# On the prevention of kidney uptake of radiolabeled DARPins

**DOI:** 10.1186/s13550-020-0599-1

**Published:** 2020-02-04

**Authors:** Mohamed Altai, Javad Garousi, Sara S. Rinne, Alexey Schulga, Sergey Deyev, Anzhelika Vorobyeva

**Affiliations:** 1 Department of Immunology, Genetics and Pathology, Uppsala University, SE-75185 Uppsala, Sweden; 2 Department of Medicinal Chemistry, Uppsala University, Uppsala, Sweden; 3 Shemyakin-Ovchinnikov Institute of Bioorganic Chemistry, Russian Academy of Sciences, Moscow, Russia; 4 National Research Tomsk Polytechnic University, Tomsk, Russia; 5 Center of Biomedical Engineering, Sechenov University, Moscow, Russia

**Keywords:** DARPin, Radiolabel, Kidney, Reabsorption, Renal uptake

## Abstract

**Background:**

Designed ankyrin repeat proteins (DARPins) are small engineered scaffold proteins (14–18 kDa) that demonstrated promising tumor-targeting properties in preclinical studies. However, high renal accumulation of activity for DARPins labeled with residualizing labels is a limitation for targeted radionuclide therapy. A better understanding of the mechanisms behind the kidney uptake of DARPins could aid the development of strategies to reduce it. In this study, we have investigated whether the renal uptake of [^99m^Tc]Tc(CO)_3_-G3 DARPin could be reduced by administration of compounds that act on various parts of the reabsorption system in the kidney.

**Results:**

Co-injection of lysine or Gelofusine was not effective for the reduction of kidney uptake of [^99m^Tc]Tc(CO)_3_-G3. Administration of sodium maleate before the injection of [^99m^Tc]Tc(CO)_3_-G3 reduced the kidney-associated activity by 60.4 ± 10.3%, while administration of fructose reduced it by 46.9 ± 7.6% compared with the control. The decrease in the kidney uptake provided by sodium maleate was also observed for [^99m^Tc]Tc(CO)_3_-9_29 DARPin. Preinjection of colchicine, probenecid, mannitol, or furosemide had no effect on the kidney uptake of [^99m^Tc]Tc(CO)_3_-G3. Kidney autoradiography showed mainly cortical accumulation of activity for all studied groups.

**Conclusion:**

Common clinical strategies were not effective for the reduction of kidney uptake of [^99m^Tc]Tc(CO)_3_-G3. Both fructose and maleate lower the cellular ATP level in the proximal tubule cells and their reduction of the kidney reuptake indicates the involvement of an ATP-driven uptake mechanism. The decrease provided by maleate for both G3 and 9_29 DARPins indicates that their uptake proceeds through a mechanism independent of DARPin structure and binding site composition.

## Background

Development of targeted radiopharmaceuticals for precise diagnosis and efficient therapy of cancer requires high target selectivity, low uptake in normal organs and tissues, and fast clearance. A new class of targeting molecules, engineered scaffold proteins (ESPs), is promising agents for tumor-targeted delivery of radionuclides [1]. Several types of ESPs, such as affibody molecules [2], ADAPTs [3], and DARPins [4–9] demonstrated efficient tumor targeting and high contrast of radionuclide imaging in preclinical studies. DARPins are built of 4–6 repeat modules of 33 amino acids and have a molecular weight of 14–18 kDa [10]. Their small size and high affinity enable good extravasation, deep penetration, and high accumulation and retention in tumors. Due to the rapid clearance from the blood and normal tissues, they provide high imaging contrast several hours after injection and could be potentially suitable for tumor-targeted radionuclide delivery. However, similarly to other ESPs, DARPins have a high accumulation of activity in the kidneys. Having molecular weight lower than 60 kDa, DARPins are efficiently filtered through the glomerular membrane into the primary urine, and then reabsorbed and internalized by the proximal tubule cells. After proteolytic degradation in lysosomes, radiolabeled catabolites either diffuse out of the cells back to the blood (non-residualizing labels) or remain inside the cells (residualizing labels). Typically, radiometals (e.g., lutetium-177, yttrium-90, actinium-225) attached to a targeting molecule by macrocyclic chelators provide residualizing labels. Radiohalogens (e.g., iodine-131) attached directly to tyrosine or by halobenzoate linkers to lysine provide non-residualizing labels, although some residualizing radiohalogen labels have been developed [11].

For targeted radionuclide therapy, it is necessary to deliver a high local absorbed dose to tumors and low absorbed doses to normal tissues to be effective and safe. As radiolabeled targeting molecules are taken up not only in tumors but also in excretory organs, high tumor-to-normal organ ratios are required. Retention of activity in tumors and in normal organs depends on the rate of receptor internalization and residualizing properties of the label. In the case of rapid internalization in tumors, residualizing labels are preferable, as they provide longer retention of activity and higher absorbed dose to the tumor than non-residualizing labels [11]. However, internalization of ESP such as affibody molecules, ADAPTs, and DARPins is relatively slow, and typically less than 20% are internalized during 24 h [2, 3, 6]. In this case, good tumor retention is mainly dependent on high affinity to a molecular target, and residualizing properties of a label are less critical for good retention of activity in tumors. The internalization in the kidneys is always rapid and may lead to high renal accumulation of activity in the case of efficient reabsorption of a targeting protein or peptide and the use of a residualizing label. This puts a limit on the amount of activity administered to a patient. In the case of non-residualizing labels for ESP, the renal activity decreases rapidly due to leakage of radiocatabolites [2, 3, 6, 9].

Targeted radionuclide therapy using radiometal-labeled peptides or small proteins is mainly limited by radionephrotoxicity. In order to deliver the required dose to tumors without exceeding the limiting dose to the kidneys (23 Gy), several strategies have been developed. The use of labeling approaches that provide non-residualizing radiocatabolites, such as radioiodination [5, 6] or the use of some peptide-based chelators for rhenium radioisotopes and technetium-99m [12–17], was shown to decrease the retention of activity in the kidneys by several folds and to improve tumor-to-kidney ratios. Another effective strategy is the fusion of an ESP to an albumin-binding domain (ABD). ABD binds to serum albumin, which increases the hydrodynamic radius above the size of the glomerular filter in the kidneys and reduces glomerular filtration. This approach was not only effective for the reduction of kidney uptake but also improved the tumor uptake due to increased circulation time of the targeting protein [18, 19]. Pretargeting is another effective strategy for an overall reduction of activity uptake in normal organs [20]. Low accumulation of the radiolabeled secondary agent in the kidneys is a prerequisite for successful pretargeting. Pretargeting systems based on oligonucleotides [21, 22] and bioorthogonal chemistry [23, 24] were evaluated in vivo in preclinical studies, while avidin-biotin and bispecific antibody-hapten [25] were translated to clinical trials [26, 27].

In diagnostic imaging, high kidney uptake of activity could potentially preclude visualization of small metastasis in the peritoneal region [28]. However, a clinical study with [^111^In]In-labeled affibody showed that modern SPECT cameras allow for imaging of metastasis in the adrenal glands close to the kidneys [29]. Nonetheless, reduction in radioactivity uptake in normal organs is generally desirable in order to decrease dose to the patient.

The above-described approaches for the reduction of kidney uptake are based on modulation of physicochemical properties of a targeting protein, radiolabel, and are specifically tailored to a given radiolabeled tracer. Therefore, it is of interest to investigate whether a general pharmacological approach could be used to lower the uptake of peptide- and protein-based radiopharmaceuticals in the kidneys.

Common clinical strategies for kidney protection during peptide receptor radionuclide therapy (PRRT) with somatostatin analogs include infusion of positively charged basic amino acids (lysine, arginine) and Gelofusine [30, 31]. Gelofusine consists of succinylated bovine gelatin and is used in clinics as a plasma expander. Both lysine and Gelofusine are ligands for megalin, an endocytic receptor in the proximal tubular cells. The megalin system is involved in the rescue of peptides and proteins from the primary urine back to the blood [32]. However, not all peptides and proteins are rescued by this system. For example, it was previously shown for affibody molecules that the megalin system was not involved in their reabsorption in the kidneys [33]. On the other hand, for nanobodies, another type of small targeting proteins, megalin was shown to take part in their reuptake, which could be inhibited by co-infusion of lysine and Gelofusine [34].

A number of other compounds were additionally investigated to reduce the renal uptake of [^111^In]In-labeled somatostatin analogs. Rolleman et al. found that the administration of colchicine and fructose was effective for the reduction of kidney uptake of [^111^In]In-DTPA-octreotide [35]. Melis et al. demonstrated that sodium maleate also reduced the kidney uptake of [^111^In]In-DTPA-octreotide [36]. Stahl et al. studied the effect of probenecid, furosemide, and mannitol on renal uptake of [^111^In]In-DOTATOC [37].

These above-discussed pharmacological options to reduce reabsorption in the kidneys have not been previously investigated for DARPins. Therefore, the present study was designed to evaluate the ability of clinically used lysine and Gelofusine to inhibit the renal accumulation of radiolabeled DARPins. In order to get a better understanding of the mechanisms behind the renal uptake of radiolabeled DARPins, we also evaluated the effect of several other compounds (sodium maleate, colchicine, mannitol, furosemide, probenecid, and fructose) that were reported to interfere with the reuptake and retention of protein-based agents in the proximal tubular cells in the kidneys.

## Methods

### General

Technetium-99m was eluted as pertechnetate from the Ultra-TechneKow generator (Mallinckrodt, The Netherlands) with sterile 0.9% sodium chloride. The CRS (Center for Radiopharmaceutical Sciences) kits for the production of tricarbonyl technetium were purchased from the CRS (PSI, Switzerland). Sodium maleate, d-fructose, colchicine, probenecid, and l-lysine were purchased from Sigma (Sigma-Aldrich, USA). Furosemid (Takeda Pharma AB, Sweden), mannitol (Fresenius Kabi AB, Sweden), and Gelofusine (B. Braun Melsungen AG, Germany) were purchased as solutions for injections. The anti-human epidermal growth factor receptor 2 (anti-HER2) DARPins G3 and 9_29 were produced in *E. coli* strain BL21(DE3) as described previously [5, 6]. DARPin G3 consists of four ankyrin repeats (14 kDa), and DARPin 9_29 consists of five ankyrin repeats (18 kDa). DARPin 9_29 binds to subdomain I of the HER2 receptor, while DARPin G3 binds to subdomain IV [38]. Amino acids involved in the interaction between 9_29 and HER2:I are different from those involved in the interaction between G3 and HER2:IV [38]. Both DARPins have a hexahistidine tag at C-terminus for site-specific labeling with tricarbonyl technetium. iTLC analysis was performed using iTLC silica gel strips (Varian, Lake Forest, USA). The radioactivity distribution along iTLC strips was measured using a Cyclone phosphor system and analyzed by the OptiQuant image analysis software (both from PerkinElmer, USA). Radioactivity in the organs and tissues was measured using an automated gamma-spectrometer with a NaI (TI) detector (1480 Wizard, Wallac, Finland).

### Radiolabeling of DARPins

Site-specific radiolabeling of hexahistidine-containing 9_29 and G3 DARPins with [[^99m^Tc]Tc(CO)_3_]^+^ to obtain a residualizing label was performed as described earlier [6]. Purification of radiolabeled DARPins by size-exclusion chromatography was performed using NAP-5 columns (GE Healthcare, UK) pre-equilibrated and eluted with PBS. Radiochemical yield and purity were measured using radio-iTLC in PBS.

### Animal studies

Female NMRI mice (10 weeks old) were housed in standard conditions at 22 °C, with laboratory food and water provided ad libitum. Mice had an adaptation period of 1 week prior to the start of experiments. For biodistribution studies, 48 mice were randomized in 12 groups to include 4 animals per group. The average animal weight at the time of experiments was 24.9 ± 1.4 g. To test the effect of various compounds on the kidney uptake of ^99m^Tc-labeled DARPin G3, mice were treated with 1 compound per group according to Table 1 before administration of [^99m^Tc]Tc(CO)_3_-G3, except lysine and Gelofusine, which were co-administered with [^99m^Tc]Tc(CO)_3_-G3. The effect of maleate administration on the kidney uptake was additionally studied for DARPin 9_29 (a variant with 5 ankyrin repeats, 18 kDa). Radiolabeled DARPins G3 (60 kBq; 0.14 nmol, 2.0 μg) or 9_29 (60 kBq; 0.14 nmol, 2.5 μg) in 100 μL of 1% BSA in PBS/mouse were administered i.v. The injected amount of protein was adjusted using a corresponding non-labeled DARPin. At 4 h p.i. of ^99m^Tc-labeled DARPins, mice were anesthetized by an i.p. injection of ketamine and xylazine solution and sacrificed by heart puncture. Blood was collected with a heparinized syringe, organs were collected and weighed, and activity was measured using a gamma spectrometer. The data was corrected for decay, and percent of ID/g of sample was calculated, except for GI tract and carcass where %ID per whole sample was calculated.

**Table 1:** List of compounds that were administered to mice before injections of radiolabeled DARPins

Compound	Suggested mechanism of action in the kidney	Route of administration	Administration respective to the radiolabeled DARPin	Dose	LD_50_
Lysine	Blocks megalin ligand-binding domains	i.v.	Co-injection	1200 mg/kg	4000 mg/kg (i.p., rat)
Gelofusine	Blocks megalin ligand-binding domains	i.v.	Co-injection	160 mg/kg	n/a
Sodium maleate	Inhibits ATP-mediated endocytosis	i.v.	5 min before	480 mg/kg	600 mg/kg (i.p., rat) 3380 mg/kg (oral, rat)
Mannitol	Diuretic, reduces contact time with scavenger receptors	i.v.	5 min before	480 mg/kg	7470 mg/kg (i.v., mouse)
Furosemide	Diuretic, reduces contact time with scavenger receptors	i.v.	5 min before	3 mg/kg	800 mg/kg (i.p., rat)
Fructose	Inhibits ATP-mediated endocytosis	i.p.	5 min before	10,800 mg/kg (60 mmol/kg)	15,000 mg/kg (83 mmol/kg)
Probenecid	Reduces renal excretion of drugs by inhibiting organic anion transporter	i.p.	1 h before	24 mg/kg	1000 mg/kg (i.p., mouse)
Colchicine	Inhibits recycling of megalin by disrupting microtubules	i.p.	5 h before	1.2 mg/kg	1.6 mg/kg (i.p., mouse)

### Autoradiography

After the gamma spectrometer measurement, two pairs of kidneys were taken from each group for autoradiography. The kidneys were embedded in the OCT cryomedium, frozen at – 80 °C, cut in serial sections (30-μm thick) using a cryomicrotome, and thaw-mounted on glass slides. For the digital autoradiography, the slides with sections were put in an X-ray cassette and exposed to phosphor screens overnight. The screens were scanned on a Cyclone Storage Phosphor System at a resolution of 600 dpi and analyzed using the OptiQuant software.

## Statistical analysis

Statistical analysis of data was performed using GraphPad Prism (Prism 7 for Windows, GraphPad Software, USA). When two sets were compared (control and maleate-treated group for 9_29), an unpaired two-tailed *t* test was used to find the significant differences. For comparison of three or more sets of data, a one-way ANOVA test with Bonferroni correction for multiple comparisons was applied.

## Results

DARPins 9_29 and G3 were radiolabeled with [^99m^Tc]Tc(CO)_3_ with 52% and 57 ± 11% radiochemical yield, respectively, and purified to provide a radiochemical purity over 97%.

The effect of the administration of various compounds on biodistribution of [^99m^Tc]Tc(CO)_3_-G3 was studied in mice at 4 h p.i. Female NMRI mice were first injected with either sodium maleate, mannitol, furosemide, mannitol, probenecid, or colchicine before the injection of [^99m^Tc]Tc(CO)_3_-G3 as described in Table 1. To study the effects of lysine and Gelofusine, they were co-administered with [^99m^Tc]Tc(CO)_3_-G3. The control group received a single injection of [^99m^Tc]Tc(CO)_3_-G3.

**Fig. 1:**
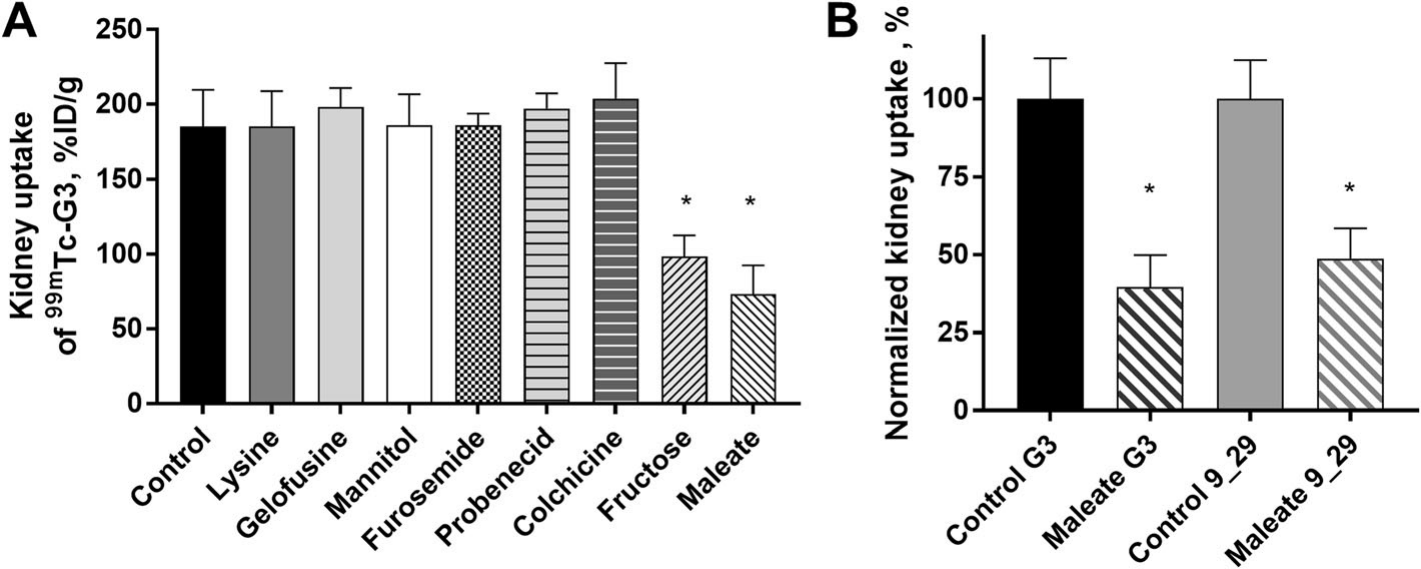
Kidney uptake of radiolabeled DARPins in female NMRI mice at 4 h pi. **a** The effect of different compounds on the kidney uptake of [^99m^Tc]Tc(CO)_3_-G3 in %ID/g. An asterisk marks a significant difference from the control (*p < 0.0001, one-way ANOVA test). Data are presented as the average of mice ± SD, for the control—as the average of eight mice ± SD. **b** The kidney uptake of both radiolabeled DARPins [^99m^Tc]Tc(CO)_3_-G3 and [^99m^Tc]Tc(CO)_3_-9_29 was decreased when maleate was administered 5 min before the DARPins. An asterisk marks a significant difference from the control (*p < 0.001, unpaired *t* test). Data are presented as average from four mice ± SD

The data for renal activity uptake after the administration of [^99m^Tc]Tc(CO)_3_-G3 is summarized in Fig. 1a. Maleate caused a selective 2.5-fold reduction of activity uptake in the kidneys (73.5 ± 19.1%ID/g) compared with the control group (185.4 ± 24.3%ID/g); however, no significant (*p* > 0.05, one-way ANOVA test) differences in the uptake in other organs or tissues were observed. Fructose administration also decreased the kidney uptake of [^99m^Tc]Tc(CO)_3_-G3 approximately two times (98.5 ± 14.0%ID/g) in comparison with the control. However, the retention of activity in other organs and the carcass was higher than in control (Table 2). The administration of colchicine did not affect the kidney uptake, but the uptake in other organs and tissues was significantly (*p* < 0.05, one-way ANOVA test) different from the control. No significant (*p* > 0.05, one-way ANOVA test) differences in the activity uptake in the blood, liver, spleen, or GI tract were observed between the control and other treatment groups.

**Table 2:** Biodistribution of [^99m^Tc]Tc(CO)_3_-G3 in NMRI mice 4 h p.i. alone (control) or after administration of compounds

	Blood	Salivary glands	Liver	Spleen	GI tract	Carcass
Control	0.33 ± 0.04	0.7 ± 0.2	3.3 ± 0.3	0.8 ± 0.2	4 ± 1	9 ± 1
Lysine	0.28 ± 0.03	0.6 ± 0.1	3.0 ± 0.2	0.6 ± 0.1	4 ± 1	7 ± 1
Gelofusine	0.29 ± 0.04	0.7 ± 0.2	2.9 ± 0.4	0.6 ± 0.1	3.4 ± 0.2	8 ± 1
Mannitol	0.31 ± 0.04	0.8 ± 0.2	3.6 ± 0.3	0.8 ± 0.1	3.9 ± 0.3	9.4 ± 0.4
Furosemide	0.33 ± 0.02	0.9 ± 0.1	3.3 ± 0.3	0.8 ± 0.1	4 ± 1	10.1 ± 0.3
Probenecid	0.32 ± 0.06	0.7 ± 0.1	3.2 ± 0.2	0.9 ± 0.1	4 ± 1	8 ± 1
Colchicine	0.44 ± 0.04*	0.5 ± 0.1	1.9 ± 0.3*	1.2 ± 0.2*	2.5 ± 0.4*	6.9 ± 0.3
Fructose	0.8 ± 0.1*	1.9 ± 0.4*	7.0 ± 1.0*	1.9 ± 0.1*	7 ± 1*	20 ± 2*
Maleate	0.33 ± 0.05	0.6 ± 0.1	3.0 ± 0.5	0.7 ± 0.2	3.4 ± 0.2	9 ± 1

Data are presented as average from four mice ± SD, for the control—as average from eight mice ± SD. Uptake is shown in %ID/g, except for GI tract and carcass, which is shown in %ID per whole sample. An asterisk marks a significant difference between the control and the treated group (*p < 0.01, one-way ANOVA test)

To study whether the effect of maleate administration on the kidney uptake was specific for the G3 DARPin scaffold, the same experiment was repeated using [^99m^Tc]Tc(CO)_3_-labeled DARPin 9_29. As shown in Fig. 1b, maleate also significantly (*p* < 0.05, t test) decreased the kidney uptake of activity compared with the non-treated control.

**Fig. 2:**
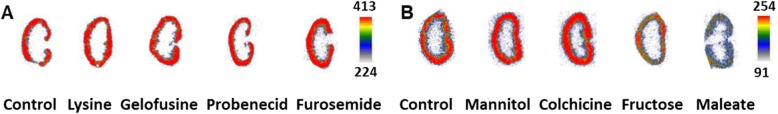
Representative ex vivo autoradiograms of the kidney sections. NMRI mice were pre- or co-injected with lysine, Gelofusine, probenecid, furosemide (**a**) and mannitol, colchicine, fructose, maleate (**b**), and [^99m^Tc]Tc(CO)_3_-G3 and sacrificed at 4 h pi. The control group was injected with [^99m^Tc]Tc(CO)_3_-G3 only

The average mouse weight was 24.9 ± 1.4 g, the average kidney weight was 264 ± 25 mg. The mice or kidney weights did not differ significantly (*p* > 0.05, one-way ANOVA test) between the groups. Representative autoradiograms of the kidney sections of mice injected with [^99m^Tc]Tc(CO)_3_-G3 from the control and treated groups are shown in Fig. 2. For all the studied groups, the activity was mainly localized in the renal cortex.

## Discussion

DARPins, a type of engineered scaffold proteins, are potentially promising vehicles for targeted delivery of radionuclides to cancer-associated molecular abnormalities. The small size of DARPins enables good extravasation into tissues, fast accumulation in tumors, and rapid clearance from the blood, providing high tumor-to-organ ratios several hours after injection. However, the majority of DARPin-associated activity is retained in the kidneys during the renal clearance. Peptides and proteins with the mass below approximately 60 kDa are efficiently filtered through the glomerular membranes and reabsorbed in proximal tubule cells of the kidney. After binding to a receptor, they are internalized by endocytosis and transported to the lysosomes [39]. In the lysosomes, the radiolabeled proteins undergo lysosomal degradation, which leads to the formation of small fragments (catabolites) [40]. Depending on the nature of the radionuclide and properties of the label, the radiocatabolites are either retained inside the cells, e.g., for residualizing labels, or excreted, e.g., for non-residualizing labels.

One way to reduce the renal retention of activity is to use non-residualizing labels. The anti-HER2 DARPins 9_29 and G3 labeled with non-residualizing iodine labels demonstrated 30 times lower activity in the kidneys compared with residualizing tricarbonyl technetium-99m labels 6 h after injection [5, 6]. The rapid internalization of DARPins and excretion of non-residualizing labels in the kidneys and liver in comparison with slow internalization in tumor cells enabled high tumor-to-organ ratios. Thus, for non-residualizing labels, the kidneys could act as a major depot of activity, releasing it back to the blood circulation. Radioiodine catabolites excreted from the kidneys to the circulation are taken up in NaI-expressing organs, e.g., thyroid and stomach. In order to prevent the accumulation of activity in these organs, we attempted to optimize the non-residualizing properties of the iodine label. In the first study, site-specific attachment of iodo-((hydroxyphenyl)ethyl) maleimide (HPEM) label via a GGGC linker indeed decreased the activity uptake in the kidneys [8]. However, a switch of renal to hepatobiliary excretion of DARPin was observed, which was undesirable. In the second study, the use of para-iodobenzoate (PIB) efficiently decreased the accumulation of activity in NaI-expressing organs without affecting the tumor targeting [9]. Due to stronger residualizing properties of the [^125^I]I-PIB-label, the retention of activity in the kidneys at 3 h p.i. was higher than that for the direct labeling; however, the renal activity rapidly decreased by 6 h p.i.

Another way to reduce the retention of activity in the kidneys, while avoiding long circulation time in the blood, is the optimization of labeling chemistry and residualizing properties of the label. Careful selection of peptide-based chelators, such as mercaptoacetyl- [12, 13, 41, 42] or cysteine-containing chelators [13, 15, 16], for labeling of affibody molecules with technetium-99 m or rhenium-188/rhenium-186 enabled up to 50-fold lower retention in the kidneys compared with the parental structures [14, 17]. However, due to the different chemical properties, many clinically promising radionuclides (e.g., ^177^Lu) cannot benefit from this approach.

For residualizing metal labels, one potential solution to the reuptake problem is to prevent glomerular filtration of the targeting protein by increasing its molecular weight. Fusion of Z_HER2:342_ and Z_HER2:2891_ affibody molecules with ABD enabled efficient reduction of the kidney uptake and the higher dose delivered to the tumor than to the kidneys with ^177^Lu [18, 19]. However, it is important to optimize the residence time of a radiolabeled conjugate in circulation to prevent irradiation of the radiosensitive bone marrow with this approach.

In this study, we have investigated whether the renal uptake of [^99m^Tc]Tc(CO)_3_-G3 DARPin could be reduced by the administration of compounds that act on various parts of the reabsorption system in the kidney.

Colchicine is an anti-gout drug that inhibits microtubule polymerization and disrupts intracellular trafficking of organelles in cells. Colchicine was shown to move megalin from the brush membrane to other intracellular compartments [43]. Administration of colchicine to rats leads to a decrease in the kidney uptake of [^111^In]In-DTPA-octreotide, which was additionally enhanced by co-injection of lysine [35].

Sodium maleate was also shown to affect the redistribution of megalin in the proximal tubules in rats [43]. Maleate reacts with succinyl-CoA, which leads to the inhibition of citric acid cycle and ATP production in tubular cells [44]. This results in inhibition of renal transport systems, including protein reabsorption in proximal convoluted tubule [45], and causes transient Fanconi syndrome [46, 47]. The administration of sodium maleate reduced the renal uptake of radiolabeled somatostatin analogs to 15% of the uptake in control [36].

Another compound that affects the ATP level in the kidneys is fructose. Parenteral administration of high doses of fructose leads to the sequestration of phosphates into metabolic intermediates and decreases ATP in the liver and kidneys [48]. A large dose of 40 mmol/kg lowered ATP by 80% in the proximal straight tubule and by 60% in the proximal convoluted tubule. Twice lower dose of 20 mmol/kg caused a similar effect, by lowering ATP in straight and convoluted tubules by 76% and 41%, respectively. Interestingly, the administration of 20 mmol/kg fructose did not affect the kidney uptake of [^111^In]In-DTPA-octreotide [35].

Probenecid is another anti-gout drug that inhibits organic anion transporter (OAT) and renal excretion of some drugs, thereby prolonging their half-life in the plasma. Stahl et al. reported that the use of probenecid reduced kidney uptake of [^111^In]In-DOTATOC by 30% [37]. That study also evaluated the effect of two types of diuretics, furosemide and mannitol, on reabsorption of [^111^In]In-DOTATOC. Furosemide acts in the distal part of the loop of Henle, and mannitol is an osmotic diuretic. Furosemide administration led to a 44% increase in renal activity accumulation of [^111^In]In-DOTATOC, while mannitol had no effect.

In this study, autoradiography demonstrated mainly cortical accumulation of activity for all studied groups, which indicates the involvement of a (proximal) tubular mechanism in the reabsorption of [^99m^Tc]Tc(CO)_3_-G3, similar to other targeting radiolabeled proteins and peptides. However, lysine and Gelofusine, which were effective for reducing the kidney uptake of somatostatin peptides and nanobodies, did not decrease the kidney uptake of DARPins. We have observed similar results earlier for another class of ESPs, affibody molecules [33]. The administration of colchicine, which inhibits the recycling of megalin receptor to the cell surface, did not reduce the uptake of DARPins. Probenecid was not effective for lowering renal activity of G3, suggesting no contribution of organic anion transport to its renal reabsorption.

Similarly to the somatostatin analogs, the uptake of radiolabeled G3 and 9_29 DARPins in the kidneys was lowered by maleate. A high dose of fructose also reduced the renal uptake of G3. Although the exact mechanism of maleate or fructose action on renal reabsorption of proteins is not described, it could be speculated that these compounds cause a disruption of energy metabolism, lowering ATP-mediated uptake and endocytosis in proximal tubule cells. As the doses of maleate and fructose used in this study were high, it would further be of interest to see if lower doses or different administration schedules could provide a similar effect. As the degree of activity reduction provided by maleate and fructose would not be sufficient for potential application of DARPins for radionuclide therapy, the most promising strategies to reduce the activity uptake in the kidneys would be their fusion to ABD or pretargeting.

## Conclusion

Common clinical strategies were not effective for the reduction of the kidney uptake of [^99m^Tc]Tc(CO)_3_-G3. Both fructose and maleate lower the cellular ATP level in proximal tubule cells, and their reduction of the kidney reuptake indicates the involvement of an ATP-driven mechanism of uptake. The decrease provided by maleate for both DARPins, G3, and 9_29, indicates that their uptake proceeds through a mechanism independent of DARPin structure and binding site composition. This knowledge could contribute to further understanding of the mechanisms behind the kidney reabsorption of radiolabeled ESPs.

## Data Availability

All data generated or analyzed during this study are included in this published article.

## Abbreviations

ATP: Adenosine triphosphate
DARPin: Designed ankyrin repeat protein
GI: Gastrointestinal
i.p.: Intraperitoneal
i.v.: Intravenous
ID/g: Injected dose per gram
iTLC: Instant thin-layer chromatography
p.i.: Post-injection
PBS: Phosphate-buffered saline
SD: Standard deviation
SPECT: Single-photon emission computed tomography
